# Influence of Nutritional Intake of Carbohydrates on Mitochondrial Structure, Dynamics, and Functions during Adipogenesis

**DOI:** 10.3390/nu12102984

**Published:** 2020-09-29

**Authors:** Manon Delcourt, Vanessa Tagliatti, Virginie Delsinne, Jean-Marie Colet, Anne-Emilie Declèves

**Affiliations:** 1 Metabolic and Molecular Biochemistry Unit, Faculty of Medicine and Pharmacy, Research Institute for Health Sciences and Technology, UMONS, 20 place du Parc, 7000 Mons, Belgium; anne-emilie.decleves@umons.ac.be; 2 Human Biology and Toxicology unit, Faculty of Medicine and Pharmacy, Research Institute for Health Sciences and Technology, UMONS, 20 Place du Parc, 7000 Mons, Belgium; vanessa.tagliatti@umons.ac.be (V.T.); virginie.delsinne@umons.ac.be (V.D.); jean-marie.colet@umons.ac.be (J.-M.C.)

**Keywords:** carbohydrates, nutrition, adipogenesis, mitochondrion, 3T3-L1 cell line, differentiation

## Abstract

Obesity is an alarming yet increasing phenomenon worldwide, and more effective obesity management strategies have become essential. In addition to the numerous anti-adipogenic treatments promising a restauration of a healthy white adipose tissue (WAT) function, numerous studies reported on the critical role of nutritional parameters in obesity development. In a metabolic disorder context, a better control of nutrient intake is a key step in slowing down adipogenesis and therefore obesity. Of interest, the effect on WAT remodeling deserves deeper investigations. Among the different actors of WAT plasticity, the mitochondrial network plays a central role due to its dynamics and essential cellular functions. Hence, the present in vitro study, conducted on the 3T3-L1 cell line, aimed at evaluating the incidence of modulating the carbohydrates intake on adipogenesis through an integrated assessment of mitochondrial structure, dynamics, and functions-correlated changes. For this purpose, our experimental strategy was to compare the occurrence of adipogenesis in 3T3-L1 cells cultured either in a high-glucose (HG) medium (25 mM) or in a low-glucose (LG) medium (5 mM) supplemented with equivalent galactose (GAL) levels (20 mM). The present LG-GAL condition was associated, in differentiating adipocytes, to a reduced lipid droplet network, lower expressions of early and late adipogenic genes and proteins, an increased mitochondrial network with higher biogenesis marker expression, an equilibrium in the mitochondrial fusion/fission pattern, and a decreased expression of mitochondrial metabolic overload protein markers. Therefore, those main findings show a clear effect of modulating glucose accessibility on 3T3-L1 adipogenesis through a combined effect of adipogenesis modulation and overall improvement of the mitochondrial health status. This nutritional approach offers promising opportunities in the control and prevention of obesity.

## 1. Introduction

For long considered as a passive organ only contributing to lipids homeostasis, the white adipose tissue (WAT) is considered as a central actor in the regulation of the global systemic metabolism nowadays [1,2]. The nutrient reservoir function of this tissue, which is mainly mediated through its ability to store energetical resource in the form of triglycerides, represents an ingenious evolutionary adaptation mechanism helping complex organisms cope with nutrient deprivation situations [3,4]. In addition to this function, WAT also plays an important role as an endocrine and secretory organ through the release of a family of signaling molecules known as adipokines [5,6,7]. Moreover, WAT has some extraordinary adaptative and plasticity skills to ensure its pivotal role in maintaining systemic metabolic homeostasis. Over the past decades, more attention has been paid to increasing cases of metabolic syndromes and associated obesity that are inherently linked to energetical status disturbances, themselves resulting in significant changes of WAT dynamics features [8,9,10].

At the cellular scale, the increases in both the number of adipocytes (hyperplasia) and their capacity of lipid storage (hypertrophy) are well described as contributing mechanisms to the abnormal and pathological WAT expansion seen in obese individuals [11,12,13]. In addition to this pathological condition, WAT expansion also occurs in a more physiological context during development [14,15]. Several models are currently available to better understand WAT remodeling, especially which parameters can modulate its functions and plasticity. Such models aim at reproducing the differentiation and maturation of precursor cells into adipocytes, which is a process called adipogenesis. Among them, the mouse embryonic fibroblast cell line 3T3-L1 is very prized especially for its differentiation skills into mature adipocytes [16,17]. 

At the molecular scale, it is now largely accepted that adipogenesis is a highly coordinated process requiring a finely orchestrated expression of several factors correlated to some deep overall morphological and functional changes in adipocytes [18,19,20,21]. Comparable to a building process involving successive interdependent phases, early events in adipogenesis determine the success of further adipocyte maturation. For that reason and through the adipogenic process, different critical steps follow one another, which are all triggered and rhythmed by the transcription of specific genes, proper modulations in the expression and activity of key proteins, and finally the acquisition of specific metabolic skills. 

At the subcellular scale, to ensure such complex and strongly coordinated processes, master regulators come into play. At this stage, it is obvious that mitochondria take a central position [22]. As a highly dynamic organelle, the mitochondrial network remodeling is particularly sensitive to both nutrient availability and cell energy requirement. Mitochondria, through their dominant position in the oxidative metabolism, play a critical function in cell energetical management and are therefore at the crossroad between various physiological and pathological conditions [23]. A cause and effect link between a mitochondrial dysfunction and obesity is clearly supported by the literature [24,25,26,27]. The dysfunction can affect three main features: structure, dynamics, and functions of the mitochondrial network. Hence, any change in the mitochondrial network traits is the subject of in-depth investigations, especially during physiological and pathological WAT remodeling. 

Interestingly, numerous studies have demonstrated that under pathological conditions such as the metabolic syndrome, an unbalanced energetical status, usually correlated to nutritional parameters, induces WAT remodeling. This stimulated the formulation of a “nutritional programming” concept in the context of metabolic syndrome [28,29,30]. Nutritional programming is defined as a response mechanism to nutrient quality and quantity variation during some key developmental phases of the organism, possibly leading to permanent sequelae [31]. This concept throws light on the pivotal role of both nutrient quality and availability features on cellular becoming and, in the context of a metabolic syndrome, on the onset of WAT dysfunction [32,33]. Among others, previous studies evidenced a correlation between the carbohydrate’s quantity and mitochondrial stress, showing the importance of the “availability” parameter [34]. In addition, various compositions of carbohydrate diet were evaluated to reduce insulin resistance and other systemic metabolic homeostasis consequences linked to supraphysiological glucose levels and their associated glucotoxic effects in several organs [35,36,37,38]. Among them, the WAT, which is known as a huge glucose consumer and storage tissue, is sensitive to environmental carbohydrates levels to promote remodeling mechanisms observed in an obesity context [39,40,41]. In an interesting way, 3T3-L1 adipogenesis was demonstrated to be influenced by the carbohydrate’s quality parameter, indicating how crucial this nutritional factor can be [42]. More recently, a partial replacement of glucose by galactose was proposed as a preventing mechanism to develop adiposity later in life, demonstrating the importance of the “quality” parameter [43,44]. In such a context, deeper investigation of the exact and specific impact of both lowering and replacing glucose by galactose strategy on the adipogenesis process should be pursued. 

In the present study, the influence of carbohydrates intake on adipogenesis was specifically and precisely investigated in differentiating 3T3-L1 cells from the angle of mitochondrial features. 

## 2. Materials and Methods 

### 2.1. Cell Culture and Differentiation 

**Figure 1:**
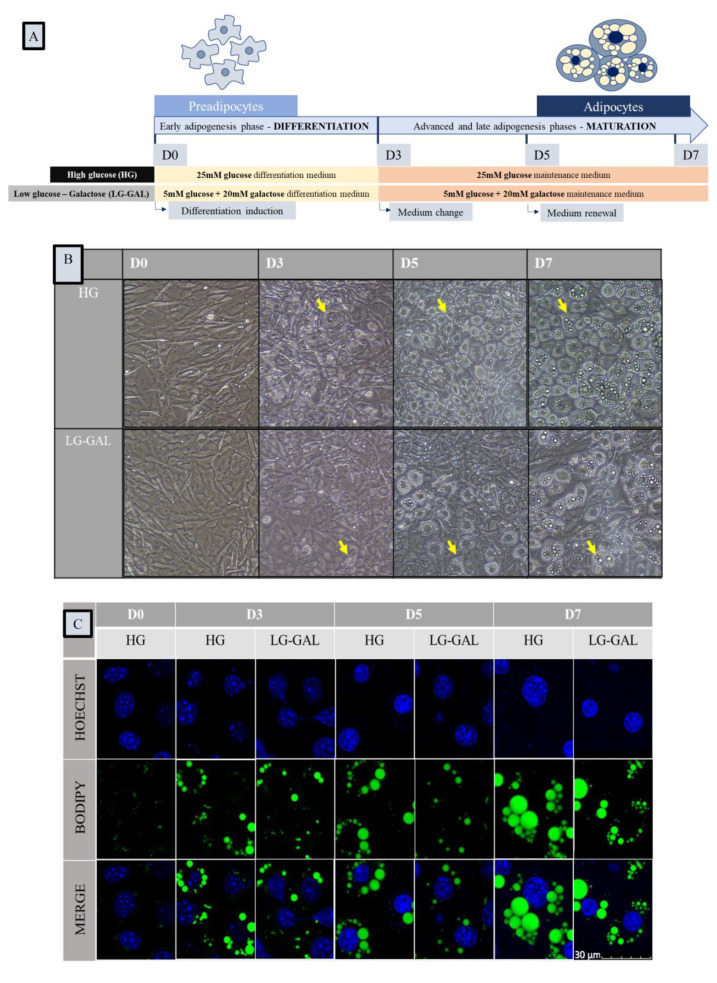

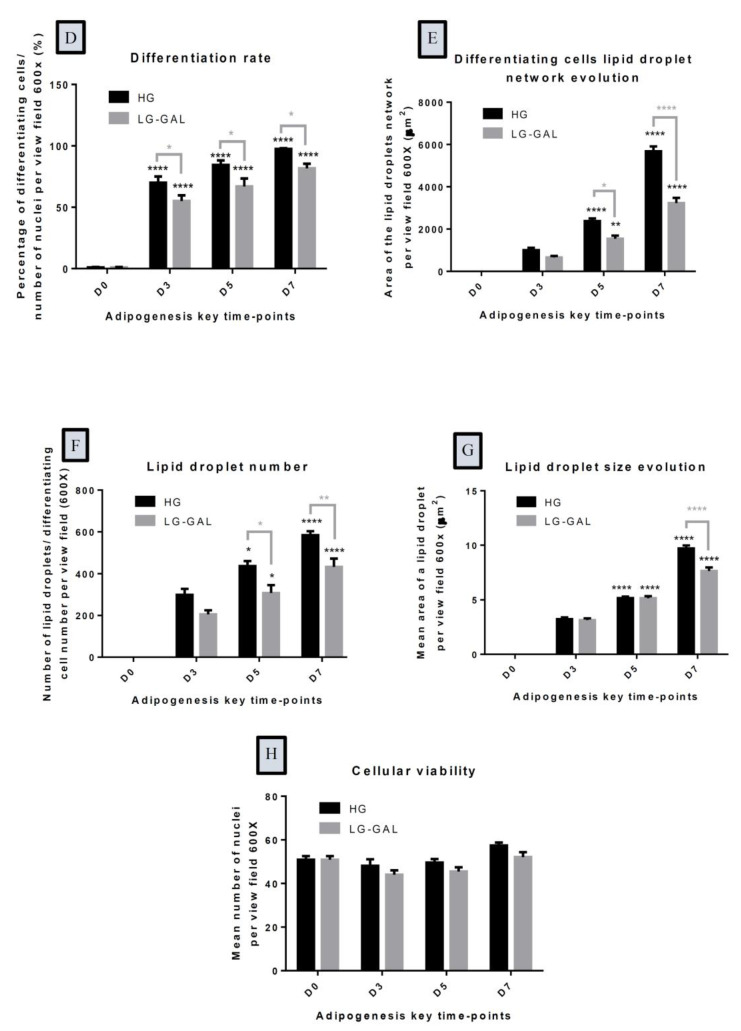
Carbohydrates quality and quantity impact adipocytes morphology and lipid droplets network evolution but not cellular viability during adipogenesis. (**A**): Schematic representation of 3T3-L1 cells differentiation experimental design in high glucose (HG) vs. low glucose–galactose (LG-GAL) conditions; (**B**): Representative bright-field micrographies of 3T3-L1 cells at the several key time points of adipogenesis in HG vs. LG-GAL conditions. Yellow arrow indicates differentiating cells; (**C**): Representative fluorescent micrographies of the lipid droplets network of 3T3-L1 cells at the several key time points of adipogenesis in HG vs. LG-GAL conditions. Hoechst staining evidences nuclei in blue, Bodipy staining evidences lipid droplets in green; (**D**): Percentage of differentiating cells at the several key time points of adipogenesis in HG vs. LG-GAL conditions. Results are the mean ratio of the number of differentiating 3T3-L1 cells to the total number of nuclei per view field; (**E**): Lipid droplet network area per view field (600×) at the several key time points of adipogenesis in HG vs. LG-GAL conditions; (**F**): Mean number of lipid droplets per differentiating 3T3-L1 cell per view field (600×) at the several key time points of adipogenesis in HG vs. LG-GAL conditions; (**G**): Mean lipid droplet area at the several key time points of adipogenesis in HG vs. LG-GAL conditions; (**H**): 3T3-L1 cells viability determined by the number of nuclei per view field at the several key time points of adipogenesis in *HG* vs. *LG-GAL* conditions. Figure D–H are the means ± SEM for n = 15 view fields (600×). Statistical (n.s, * *p* < 0.05; ** *p* < 0.01; **** *p* < 0.0001) comparison of each key time points to the D0 situation (*in black*) and between HG vs. LG-GAL (*in gray*) were performed by two-way ANOVA and Holm–Sidak’s multiple-comparison test.

Murine 3T3-L1 preadipocytes were purchased from the American Type Culture Collection (ATCC) and sub-cultured in growth media consisting of Dulbecco’s Modified Eagle’s Medium (DMEM) high glucose (HG) (4.5 g/L glucose, Sigma-Aldrich, St. Louis, MO, USA) supplemented with 10% Fetal Bovine Serum (FBS) premium (PanBiotech, Aidenbach Germany)). At passage 7 (P7), cells were cultured until 100% confluence and then let for two additional days before inducing differentiation (D0). The experimental scheme of 3T3-L1 differentiation in our study is illustrated in Figure 1A. Briefly, on D0, cells were exposed during 3 days to a differentiation medium (DM) consisting of culture medium + 10% FBS + an adipogenic cocktail (Insulin 1 mg/mL (Sigma-Aldrich, St. Louis, MO, USA), Isobutylmethylxanthine12 mg/mL (Sigma-Aldrich, St. Louis, MO, USA) and Dexamethazone 0.4 mg/mL (Sigma-Aldrich, St. Louis, MO, USA)). The culture medium was different depending on the condition: DMEM HG (25 mM glucose, Sigma-Aldrich, St. Louis, MO, USA) was used for the HG condition, a DMEM low glucose (LG) (5 mM glucose, Sigma-Aldrich, St. Louis, MO, USA) was used for the LG condition, and a DMEM LG (5 mM glucose, Sigma-Aldrich, St. Louis, MO, USA) + GAL (20 mM galactose, Sigma-Aldrich) was used for the LG-GAL condition. On D3, the DM was replaced by a maintenance medium (MM) containing the same components as DM except IBMX and DEX. MM was replaced every two days until D7, a time point to which a mature adipocytes state is reached. Cells were harvested on D0, D3, D5, and D7 to perform the following analyses. The medium of cells was sampled for carbohydrates levels assessment before being changed or renewed. 

### 2.2. Cellular Morphology Assessment Using Bright Field Microscopy 

Micrographs of 3T3-L1 cells differentiated in either LG-GAL or HG in T75 flasks were taken all along the differentiation process on D0, D3, D5, and D7. Images were acquired on a Nikon Eclipse i20 microscope. 

### 2.3. Lipid Droplets and Mitochondrial Networks Assessment Using Fluorescence Microscopy 

Three different fluorescent probes were used: BODIPY 493/503 (5 mM stock solution, Invitrogen) to stain the lipid droplet network, Mitotracker Red CMXRos (Molecular Probes, Eugene, OR, USA) to stain the mitochondrial network, and Hoechst 33.342 (Miltenyi Biotec GmbH Bergisch Gladbach) to stain the nuclei. On D0, D3, D5, and D7, cells were incubated for 15 min at 37 °C with a staining solution containing the three fluorescent probes. After the incubation period, living cells images were done using a confocal microscope (OLYMPUS FV1000 Confocal) with 60× (oil) objective (MitoTracker Red λ excitation: 579 nm, λ emission: 599 nm; BODIPY λ excitation: 495 nm, λ emission: 519 nm, Hoechst λ excitation: 352 nm, λ emission: 461 nm). Images were captured with a scanning speed of 12.5 pixels/s, image resolution of 1024 × 1024 pixels, and then analyzed using ImageJ software. Parameters regarding the lipid droplet network and the number of differentiated cells were further analyzed using MRI_Lipid Droplets tool (a macro of ImageJ). Each intensity or area measurement was normalized to the number of differentiated cells and further analyzed using Excel functionalities (Microsoft**^®^**). 

### 2.4. Carbohydrates Levels Assessment 

Extracellular carbohydrates levels were determined on culture media on D0, D3, D5, and D7 of differentiating 3T3-L1 cells. Intracellular carbohydrates levels were also measured on D0, D3, and D7 on 3T3-L1 using a chloroform–methanol–water extraction method separating the cell content into two main phases [37]. The upper phase (methanol–water phase) containing hydrophilic metabolites was collected, evaporated with a speed vacuum, and resuspended using phosphate buffer, pH 7.4. Once the metabolites resuspended, the solution was mixed with trimethylsilylpropanoic acid (TSP) at a final concentration of 1 mM and transferred in 5 mm diameter NMR tubes. ^1^H NMR spectra of the intracellular aqueous phase and extracellular fluids were acquired on a Bruker Advance 600 MHz spectrometer with a 5 mm PABBO BB-probe. A NOESYPRESAT-1D sequence was used with 256 scans. The acquired FIDs (Free Induction Decays) were Fourier transformed to obtain spectra. Mestre Nova 11 program ((Mestrelab Research) was used to perform spectra analyses. Area under the curve (AUC) values obtained performing “peak picking” (analytical tool on the program Mestre Nova 11) and corresponding to the glucose (5.25 ppm) and galactose (5.27 ppm) peaks were used to perform a semi-quantitative carbohydrates levels assessment. 

### 2.5. Protein Expression: Immunoblotting Analysis 

First, 3T3-L1 cells were homogenized and lysed using cell lysis buffer (CST) supplemented with protease inhibitor cocktail (Sigma-Aldrich, St. Louis, MO, USA) and 1 mM DTT. Protein yield was assessed using a Pierce™ BCA Protein Assay Kit (Thermo Fisher Scientific, Waltham, MA, USA). The cell lysates corresponding to 30 μg proteins were resolved by electrophoresis on 12% precast Bis-Tris gel (Bio-Rad Laboratories, Hercules, CA, USA), and proteins were transferred from the gel to a nitrocellulose membrane (GE Healthcare Europe GmbH). Protein transfer was confirmed through Red Ponceau staining. The blot was blocked with 5% non-fat dry milk diluted in Tris-Buffered Saline, 0.1% TWEEN**^®^** 20 Detergent (1× TBST) for 1 h at room temperature. Then, the membranes were incubated at 4 °C overnight with primary antibodies in TBST 0.5% bovine serum albumin (BSA). The following antibodies and dilutions were used, and interest proteins were detected by immunoblotting using primary antibodies at the following dilutions: 1:1000 anti-perilipin-1 (Cell Signaling Technology, Danvers, MA, USA), 1:500 anti-adiponectin (Thermo Fisher Scientific, Waltham, MA, USA), 1:1000 anti-Dynamin-1-like protein (Anti-Drp1) (Cell Signaling Technology, Danvers, MA, USA), 1:1000 anti-Mitofusin2 (Cell Signaling Technology, Danvers, MA, USA), 1:1000 anti-fumarase (Cell Signaling Technology, Danvers, MA, USA), and 1:1000 anti-SdhA (Cell Signaling Technology, Danvers, MA, USA). Horseradish peroxidase (HRP)-conjugated anti-rabbit IgG (Immunoglobulin G) at 1:10,000 dilution was used as secondary antibody (GE Healthcare, Little Chalfont, UK). Blots were revealed with SuperSignal™ West Dura Extended Duration Substrate (Thermo Fisher Scientific, Waltham, MA, USA) and scanned using C-DiGit **^®^** Blot Scanner (LI-COR Biosciences, Lincoln, NE, USA). Densitometric analyses were performed using Image Studio Lite software (LI-COR biosciences). 

### 2.6. Gene Expression: RT-qPCR Analysis

First, 3T3-L1 cells were homogenized in Trizol, and total RNA was purified using RNeasy columns (Qiagen Hilden, Germany) at the different key time points (D0, D3, D5, and D7). RNA was reverse transcribed to cDNA using the Maxima First Strand cDNA synthesis KIT (Thermo Fisher Scientific, Waltham, MA, USA) in a qPCR Instrument (Roche). Gene expression was analyzed by Real-Time quantitative PCR (RT-qPCR) using LightCycler FastStart DNA Master SYBR Green I kit (Roche Diagnostics; Indianapolis, IN) and a LightCycler real-time thermal cycler (Roche Diagnostics). The PCR conditions were 95 °C for 10 min (1 cycle); 95 °C for 15 s; and 60 °C for 1 min (40 cycles). PPAR-γ, PREF-1, PLIN1, FASN, ADIPOQ, PGC-1α, SDHA and FH gene expressions were evaluated at the different key time points. The primer sequences used in the study are available on request. Data were analyzed using Lightcycler 96 Software (Roche). Relative gene expressions were calculated using the 2^-ΔΔCt^ method.

### 2.7. Statistical Analysis

Data from at least three independent experiments (n = 3, biological triplicate) were analyzed using two-way ANOVA and Holm–Sidak’s multiple-comparison test. Statistical analyses were performed with GraphPad Prism 6 software. Results are presented as mean values ± SEM. The level for statistical significance was defined as *p* < 0.05 or less. Confidence intervals of 95% were automatically determined, and effect sizes were assessed by checking the variance explained. 

## 3. Results

### 3.1. Carbohydrates Intake Impact Adipocyte Differentiation and Lipid Droplet Network Evolution but Not Cellular Viability during Adipogenesis

To study the impact of carbohydrates supplies on the adipogenesis and related mitochondrial remodeling, a 3T3-L1 differentiation scheme was modulated in terms of glucose and galactose intake all along the differentiation process (Figure 1A). In order to confirm that culturing 3T3-L1 cells in a strict LG condition is associated to a poorer differentiation yield, data were acquired in terms of 3T3-L1 morphological assessment in LG vs. LG-GAL vs. HG conditions (Supplementary data 1). Those data highlighted that LG is associated with a drastically lower adipogenic yield, justifying restricting our analyses to the comparison of two equimolar conditions in terms of carbohydrates exposure. Therefore, during the adipogenic process, which is usually subdivided into a differentiation and a maturation phases, 3T3-L1 cells were differentiated either in a high-glucose (HG, 25 mM glucose) or a low-glucose medium (5 mM glucose) but complemented with galactose (20 mM galactose) to ensure on one side equal carbon equivalent concentrations of 25 mM carbohydrates and on the other side equivalent osmolarity in both conditions. Cells were harvested on selected key time points (D0, D3, D5 and D7) corresponding to media changes or renewal steps. The differentiation of pre- into mature adipocytes induces some significant morphological acquisitions, including a new cellular round-shaping aspect and the formation and expansion of a lipid droplet network. Those morphological changes during differentiation were confirmed by microscopy under both carbohydrates’ conditions, however, with a poorer adipogenic yield in the LG-GAL condition (Figure 1B). Those observations are accompanied by a lower percentage of differentiating and mature cells (already statistically different on D3) during the early adipogenesis phase (Figure 1C). The phenotype of mature 3T3-L1 adipocytes is confirmed by the expansion and formation of a lipid droplet network reflected by BODIPY fluorescent staining, which clearly unveils different lipid behaviors through adipogenesis in LG-GAL and HG conditions (Figure 1D,E). Features of the lipid droplet network were further analyzed in terms of number and size parameters. As compared to the LG-GAL condition, the differentiation process in HG conditions obviously follows a significantly sharper increase of both lipid droplets number and size during advanced adipogenic phase by D5 (Figure 1E,G). Since the number of differentiating cells is expected to remain constant after the first 24 h of the mitotic clonal expansion phase, the impact of either HG or LG-GAL conditions on cell viability was assessed by counting the number of nuclei at D0 and D3, D5, and D7 of adipogenesis (Figure 1H). It appears that modulating the carbohydrates intake has no impact on cell viability during adipogenesis (Figure 1H). 

### 3.2. Carbohydrates Intake Impacts the Early Adipogenesis Phase and Consequently Performances of Mature Adipocytes

**Figure 2:**
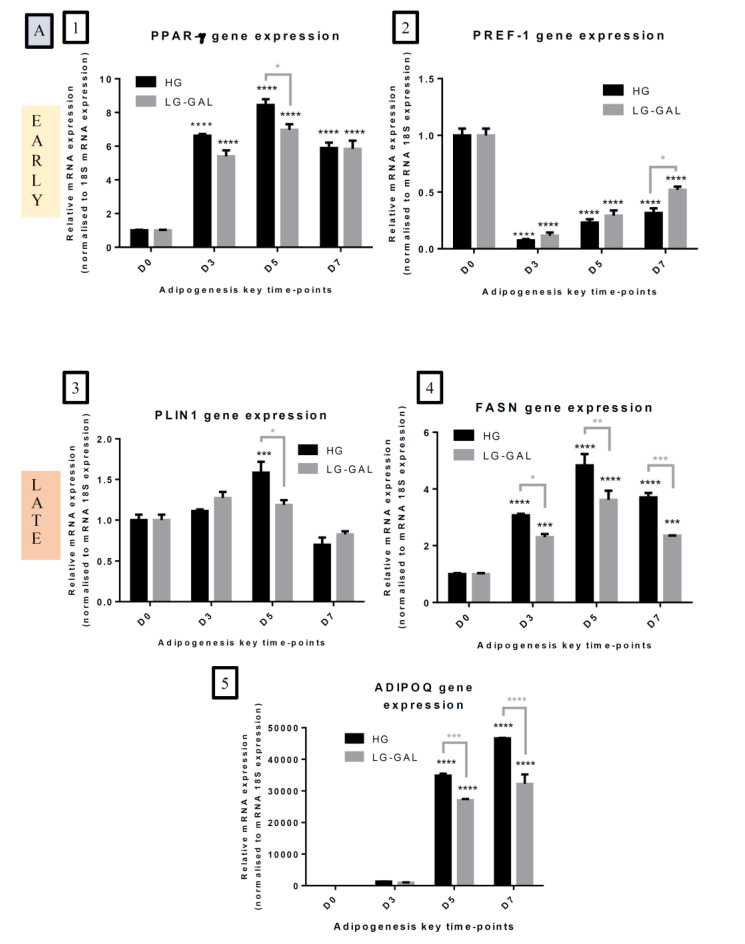

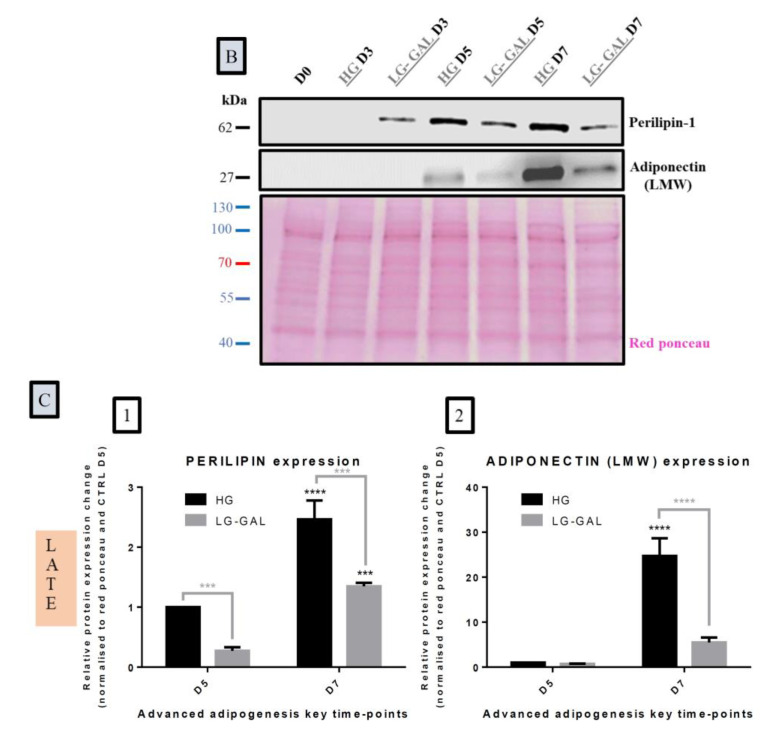
Carbohydrates Intake Impacts the Early Adipogenesis Phase and Consequently Performances of Mature Adipocytes (**A**): Early and late adipogenic markers gene expression in 3T3-L1 cells at differentiation (D0) and day 3 (D3), D5, and D7 of adipogenesis in HG vs. LG-GAL conditions. mRNA levels are relative fold changes normalized by 18 s mRNA levels and by gene of interest expression levels at D0. 1. *Peroxisome proliferator-activated receptor gamma* (PPAR-γ), 2. *Preadipocyte factor 1* (PREF-1), 3. *Perilipin-1* (PLIN1), 4. *Fatty acid synthase* (FASN), 5. *Adiponectin* (ADIPOQ); Results are the means ± SEM (biological (n = 3) and technical (n = 3) triplicates). Statistical (n.s, * *p* < 0.05; ** *p* < 0.01; *** *p* < 0.001; **** *p* < 0.0001) comparison of each time points to D0 (*in black*) and between HG vs. LG-GAL (*in gray*) were performed by two-way ANOVA and Holm–Sidak’s multiple-comparison test; (**B**): Representative immunoblot of adipogenic proteins expressed in 3T3-L1 cells during the adipogenesis maturation phase; (**C**): Protein expression of mature adipocyte markers in 3T3-L1 cells at D0 and D3, D5, and D7 of adipogenesis in HG vs. LG-GAL conditions. Data are densitometric analysis normalized by Red Ponceau densitometric values and by protein expression levels at D5 in the HG condition. 1. *Perilipin-1* protein expression; 2. *Low molecular weight* (LMW) *adiponectin* protein expression. Results are the means ± SEM (biological (n = 3) and technical (n = 3) triplicates). Statistical (n.s, * *p* < 0.05; ** *p* < 0.01; *** *p* < 0.001; **** *p* < 0.0001) comparison of each time points to the D5 situation (*in black*) and between HG vs. LG-GAL (*in gray*) were performed by two-way ANOVA and Holm–Sidak’s multiple-comparison test.

In order to confirm and validate the morphological changes and emergence of a lipid droplet network over time, key markers of both early and late adipogenesis phases were assessed in terms of gene and protein expressions. Differentiation induction is related to a significant increase at the transcriptional level of the adipogenesis master regulator, peroxisome proliferator-activated receptor γ (PPARγ), during the early adipogenesis phase yet significantly lower in the LG-GAL condition (Figure 2(A1)). In parallel, the expression of PREF-1, an autocrine and paracrine factor preventing the advancement to mature adipocytes [38,39,40], has been evaluated at the transcriptional level in both conditions. The results show a significant decrease of this adipogenesis repressor in both conditions, although to a lesser extent in the LG-GAL condition (Figure 2(A2)). At a later phase of the differentiation process, perilipin 1 (PLIN1), a gene encoding a protein surrounding a lipid droplet and playing a critical role in adipocyte lipid metabolism [45,46,47,48,49], was also assessed. By D5, a peak of PLIN1 expression was reached in the HG condition in maturating 3T3-L1 (Figure 2(A3,B,C)). After considering lipid droplets shaping and dynamic aspects, the lipogenesis enzymatic angle has been investigated through the evaluation of FASN gene expression, which is a gene encoding an enzyme catalyzing a critical step of fatty acid synthesis (Figure 2(A4)). A progressive increase of FASN expression with adipogenesis induction was noticed, yet with a lower expression level in the LG-GAL condition in differentiating 3T3-L1. In parallel, adiponectin, a major adipokine secreted by mature adipocytes, was also explored through possible changes in gene and protein expression in both conditions (Figure 2(A5,B,C2)). As for other mature adipocyte markers, adiponectin expression is significantly stimulated during more advanced adipogenesis phases in both conditions, although to a lower extent in the LG-GAL condition. 

### 3.3. Adipogenesis Progression Requires Important and Increasing Monocarbohydrates Cellular Intake

**Figure 3:**
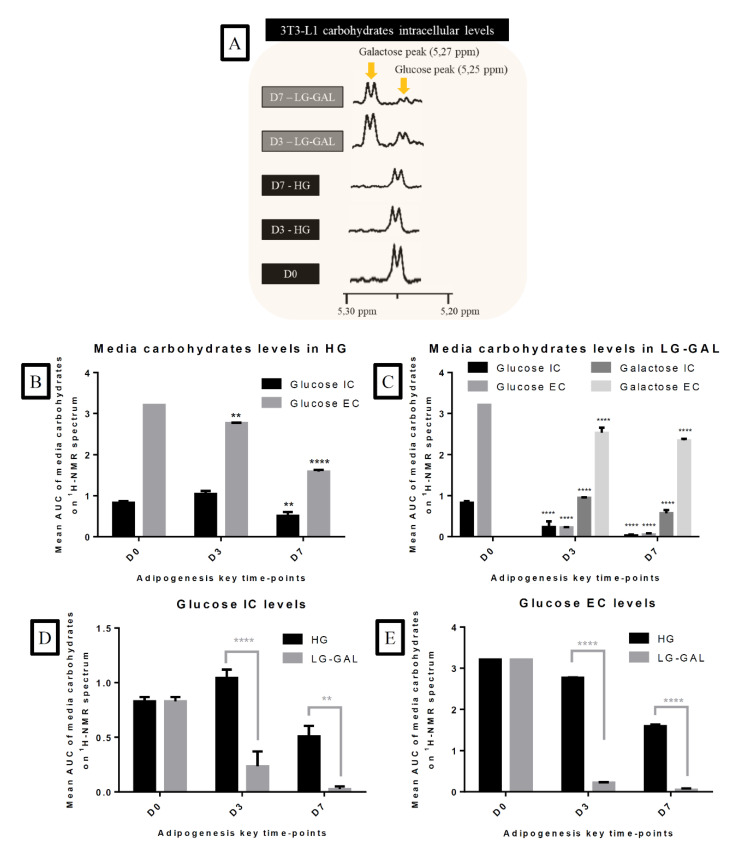
Adipogenesis Progression Requires Important and Increasing Monocarbohydrates Cellular Intake (**A**): Zoom on glucose and galactose peaks on representative ^1^H NMR spectra of intracellular 3T3-L1 cells and media at D0 and D3 and D7 of adipogenesis in HG vs. LG-GAL conditions; (**B**): Glucose intracellular (IC) and extracellular levels (EC) of 3T3-L1 cells in the HG condition; (**C**): Galactose and glucose intracellular and extracellular levels of 3T3-L1 in the LG-GAL condition. Figure B,C: Results are the mean AUC of the different peaks corresponding to either glucose or galactose on ^1^H NMR spectra of intracellular medium (n = 3); (**D**): Glucose intracellular levels of 3T3-L1 cells in HG vs. LG-GAL conditions; (**E**): Glucose extracellular levels of 3T3-L1 cells in HG vs. LG-GAL conditions. Results are the means ± SEM (biological (n = 3) triplicates). Statistical (n.s, ** *p* < 0.01; **** *p* < 0.0001) comparison of each time points to the D0 situation (in black) and between HG vs. LG-GAL (in gray) were performed by two-way ANOVA and Holm–Sidak’s multiple-comparison test.

In order to evaluate carbohydrates management and intake in differentiating and maturating 3T3-L1 adipocytes in HG vs. LG-GAL conditions, carbohydrates levels were assessed in intracellular (IC) and extracellular (EC) compartments. An enlarged view of glucose/galactose peaks in ^1^H NMR spectra of 3T3-L1 intracellular media indicates the intake of both glucose and galactose in the LG-GAL condition and high IC glucose levels in the HG condition (Figure 3A). Semi-quantitative assessment based on the integration of the area under the curve (AUC) of glucose or galactose peaks also revealed different carbohydrates supply management depending on the condition. In the HG condition, extracellular glucose levels significantly decreased all along the differentiation without reaching a zero level (Figure 3B). Similar IC and EC glucose levels evolution in 3T3-L1 are observed in the LG-GAL condition (Figure 3B,C) with a significant decrease of intracellular glucose levels on D7 occurring in both conditions. In the LG-GAL condition, galactose resources are also correlated to prevalent and increasing intake over the differentiation process as evidenced by galactose IC level increase and extracellular decrease. To evaluate if galactose intake was linked to insufficient glucose extracellular availability over the adipogenic process, both IC and EC glucose levels in both conditions were compared (Figure 3D,E). Although both IC and EC glucose levels were significantly lower in the LG-GAL condition, levels never reached an undetectable threshold.

### 3.4. The Mitochondrial Network Evolution Depends on Carbohydrates Supplies and on the Lipid Droplet Network Evolution during Adipogenesis

**Figure 4:**
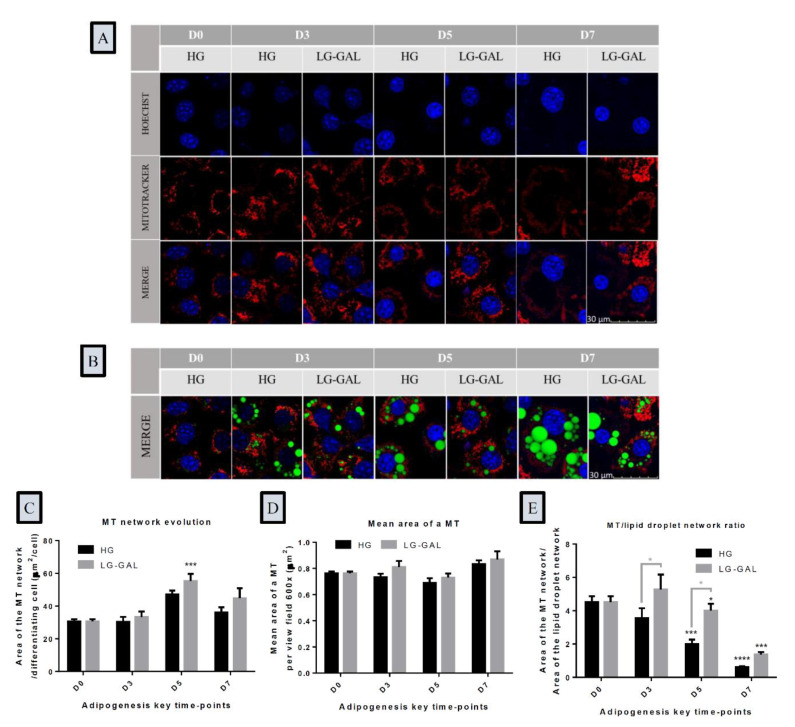
The Mitochondrial Network Evolution Depends on Carbohydrates Supplies and on the Lipid Droplet Network Evolution during Adipogenesis (**A**): Representative fluorescent micrographs of the mitochondrial (MT) network of 3T3-L1 cells at D0 and D3, D5, and D7 of adipogenesis in HG vs. LG-GAL conditions. Hoechst staining evidences nuclei in blue, MitoTracker™ Red CMXRos staining evidences the mitochondrial network in red; (**B**): Lipid droplets (BODIPY, green) and mitochondrial (MitoTracker™ Red CMXRos) network co-detection on representative fluorescent micrographs of 3T3-L1 cells in HG vs. LG-GAL conditions; (**C**): Mitochondrial (MT) network area per differentiating 3T3-L1 cell per field (600×) in HG vs. LG-GAL conditions; (**D**): Mean mitochondrion (MT) area at D0 and D3, D5, and D7 of adipogenesis in HG vs. LG-GAL conditions. (**E**): Mean mitochondrion (MT) to lipid droplet network area ratio at D0 and D3, D5, and D7 of adipogenesis in HG vs. LG-GAL conditions. Figure C–E are the means ± SEM for n = 15 view fields (600×). Statistical (n.s, * *p* < 0.05; *** *p* < 0.001; **** *p* < 0.0001) comparison of each time points to the D0 situation (in black) and between HG vs. LG-GAL (in gray) were performed by two-way ANOVA and Holm–Sidak’s multiple-comparison test.

The impact of carbohydrates supply on the functional mitochondrial network was next assessed. Fluorescent staining of the mitochondrial network of 3T3-L1 cells during the adipogenesis process evidenced an evolution of the mitochondrial network structure attuning cellular morphological changes due to differentiation and maturation (Figure 4A). Bodipy combined to MitoTracker™ Red CMXRos co-staining was also performed (Figure 4B). The results clearly show a rearrangement of the mitochondrial network in differentiating 3T3-L1 cells as well as its condensation around the nucleus and the sweeping lipid droplet network. Those observations are accompanied by significant changes regarding the area of the mitochondrial network with an important increase occurring in more advanced phases of adipogenesis, as shown in Figure 4C on D5. This increase is particularly evident in the LG-GAL condition with a tendency to observe a broader mitochondrial network over adipogenesis in 3T3-L1 cells. On the other hand, the mean mitochondrial area remained stable without any impact by carbohydrates supply (Figure 4D). Finally, the assessment of the ratio between the mitochondrial and the lipid droplet network area during adipogenesis evidenced an inverse correlation between the mitochondrial network and the lipid network expansion during adipogenesis and significantly higher MT/lipid droplet network ratios in 3T3-L1 differentiated in LG-GAL compared to the HG condition (Figure 4E). 

### 3.5. Both Mitochondrial Biogenesis and Dynamics Phenomena Are Sensitive to Adipogenesis Triggering and are Impacted by Carbohydrates Supplies

**Figure 5:**
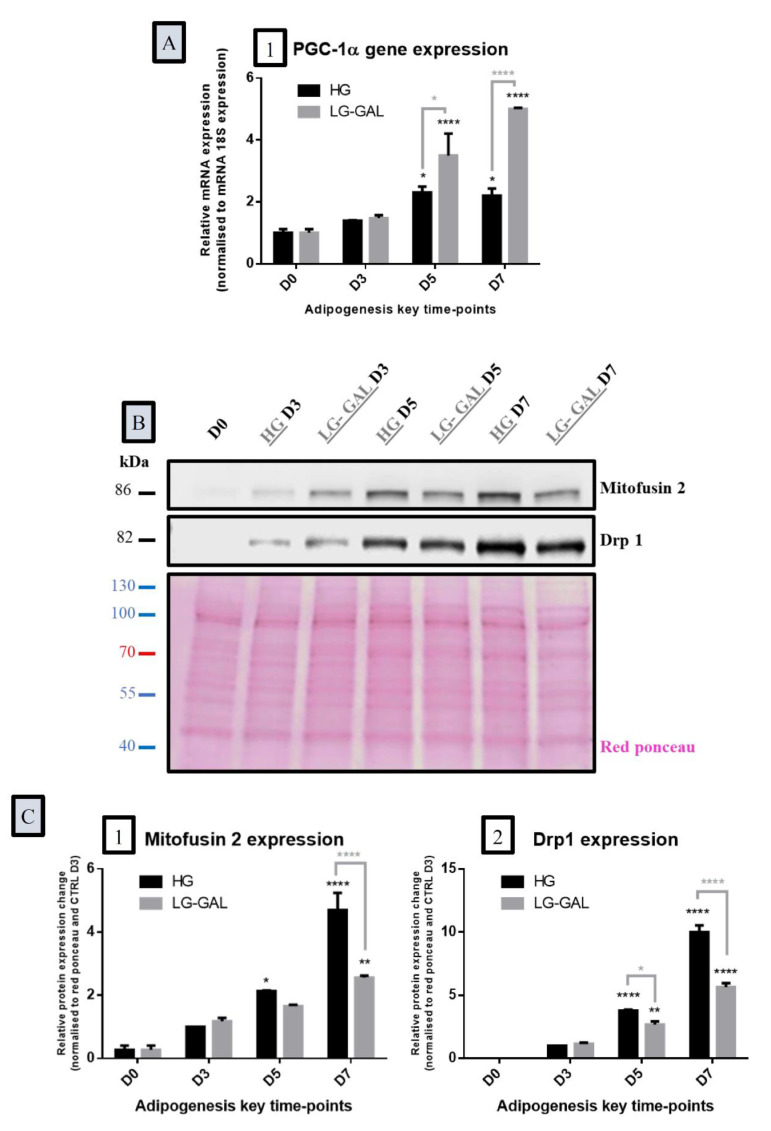
Both Mitochondrial Biogenesis and Dynamics Phenomena are Sensitive to Adipogenesis Triggering and are Impacted by Carbohydrates Supplies (**A**): Peroxisome proliferator-activated receptor gamma coactivator 1-alpha (PGC1-α) gene expression in 3T3-L1 cells at the several time points of adipogenesis in HG vs. LG-GAL conditions. mRNA levels are relative fold changes normalized by 18 s mRNA levels and by gene of interest expression levels at D0; (**B**): Representative immunoblot of mitochondrial dynamics and biogenesis-related factor proteins expressed in 3T3-L1 cells during the adipogenesis maturation phase and Red Ponceau; (**C**): Mitochondrial dynamics and biogenesis-related factor protein expression in 3T3-L1 cells in HG vs. LG-GAL conditions. Data are densitometric analysis normalized by Red Ponceau densitometric values and by protein expression levels at D3 in the HG condition. 1. Mitofusin 2 (Mfn2) as a marker of mitochondrial fusion phenomena; 2. Dynamin-1-like protein (Drp1) as a marker of mitochondrial fission phenomena. Results are the means ± SEM (biological (n = 3) and technical (n = 3) triplicates). Statistical (n.s, * *p* < 0.05; ** *p* < 0.01; **** *p* < 0.0001) comparison of each key time points to the D5 situation (in black) and between HG vs. LG-GAL (in gray) were performed by two-way ANOVA and Holm–Sidak’s multiple-comparison test.

Regarding the mitochondrial biogenesis and dynamics, the assessment of the peroxisome proliferator-activated receptor gamma coactivator 1-alpha (PGC-1α) revealed a significant increase of its gene expression in more advanced adipogenesis phases (D5 and D7) in both conditions with a significantly higher increase in the LG-GAL condition (Figure 5A). The expression of selected fusion and fission proteins-related expression were also examined to assess the long-term regulation mechanism of mitochondrial dynamics. It appears that the expression of Mfn2 protein was significantly increased during the maturating phases of adipocyte phases in both conditions, with a significant increase of this process earlier, on D5, in the HG condition (Figure 5(B,C1)). Mitochondrial fission, through Drp1 protein expression changes, also appears to increase over adipogenesis with a profound and earlier decrease, on D5, of its expression in differentiating 3T3-L1 in the LG-GAL condition (Figure 5(B,C2)).

### 3.6. Differentiating Adipocyte Mitochondria Adapt their Metabolic Function in Response to Both Carbohydrates Supplies

**Figure 6:**
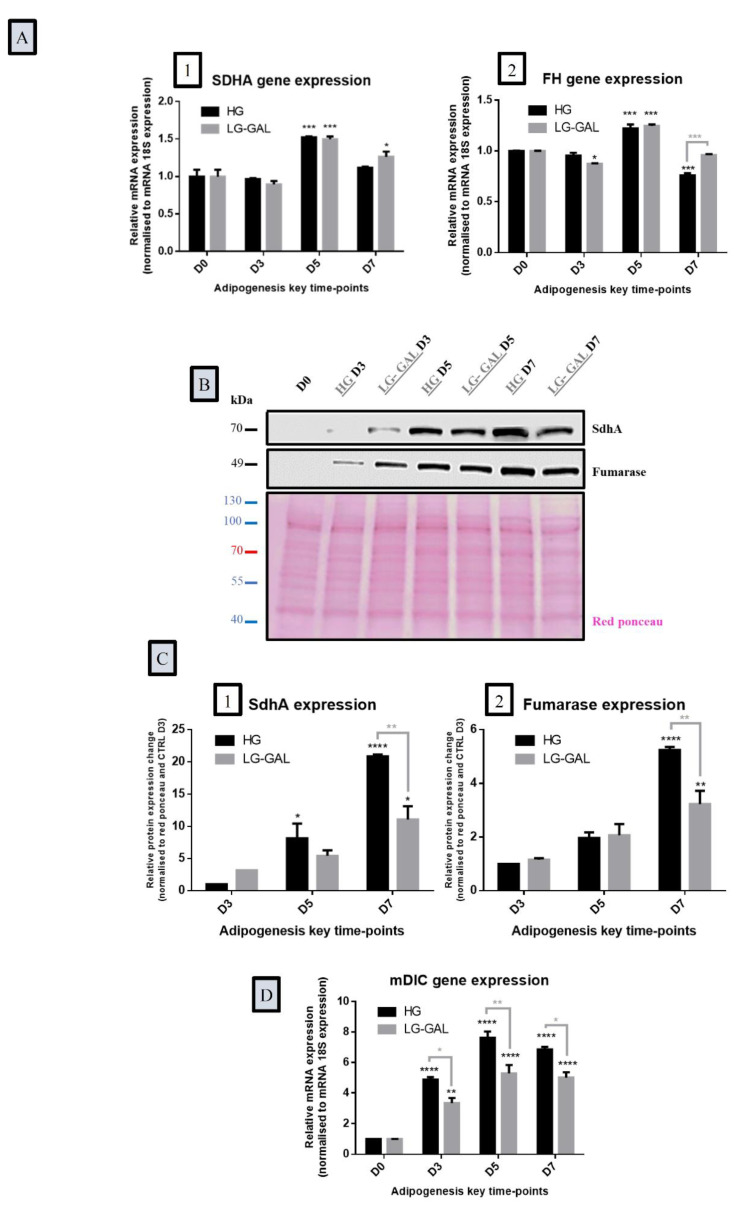
Differentiating Adipocyte Mitochondria Adapt their Metabolic Function in Response to Both Carbohydrates Supplies (**A**): Gene expression of key mitochondrial metabolic function (Krebs cycle and respiration) markers in 3T3-L1 cells at the several time points of adipogenesis in HG vs. LG-GAL conditions. mRNA levels are relative fold changes normalized by 18 s mRNA levels and by gene of interest expression levels at D0. 1. Succinate dehydrogenase subunit A (SDHA) as a marker of both Krebs cycle and mitochondrial respiration metabolism. 2. Fumarate hydratase (FH) as a marker of Krebs cycle metabolism; (**B**): Representative immunoblot of key mitochondrial Krebs cycle and respiration-related enzymes proteins expressed in 3T3-L1 cells during adipogenesis maturation phase and Red Ponceau; (**C**): Key mitochondrial Krebs cycle and respiration-related enzymes protein levels in 3T3-L1 cells in HG vs. LG-GAL conditions. Data are densitometric analysis normalized by Red Ponceau densitometric values and by protein levels at D3 in the HG condition. 1. Succinate dehydrogenase subunit A (SDHA) as a marker of both Krebs cycle and mitochondrial respiration metabolism. 2. Fumarase as a marker of Krebs cycle metabolism. Results are the means ± SEM (biological (n = 3) and technical (n = 3) triplicates). (**D**): Mitochondrial dicarboxylate carrier protein (mDIC) gene expression in 3T3-L1 cells at the several key time points of adipogenesis in HG vs. LG-GAL conditions. mRNA levels are relative fold changes normalized by 18s mRNA levels and by gene of interest expression levels at D0. Results are the means ± SEM (biological (n = 3) and technical (n = 3) triplicates). Statistical (n.s, * *p* < 0.05; ** *p* < 0.01; *** *p* < 0.001; **** *p* < 0.0001) comparison of each time points to the D5 situation (in black) and between HG vs. LG-GAL (in gray) were performed by two-way ANOVA and Holm–Sidak’s multiple-comparison test.

Finally, genes and proteins acting in cell respiration as well as mitochondrial transport systems were evaluated in both HG and LG-GAL conditions during adipogenesis. The gene expression of the succinate dehydrogenase subunit A (SDHA), a marker of both Krebs cycle and mitochondrial respiration [50], was significantly increased on D5 without noticeable differences between HG and LG-GAL conditions (Figure 6A1). Similarly, the fumarase (FH) enzyme was transcriptionally expressed during late adipogenic phases, reaching a peak of expression by D5 in both conditions. At the proteic level, both SdhA and fumarase expressions sharply increased over the adipogenic process, reaching significant increased levels on D7 in differentiating 3T3-L1 (Figure 6C1,C2). Those observations are correlated to gene expression induction on D5, subsequently leading to protein expression increase on D7. Regarding the mitochondrial transport, the gene expression of the mitochondrial dicarboxylate carrier protein (mDIC) displayed a significant increase during the adipogenesis, yet it was significantly lower in the LG-GAL condition as compared to the HG one on D3, D5, and D7 (Figure 6D).

## 4. Discussion

In both physiological and pathological contexts, adipogenesis is known to be highly sensitive to the nutritional composition. Especially, changes in the carbohydrates resources are expected to modulate this differentiation process [40,42,51,52,53]. As obesity and co-morbidities are increasing worldwide, (re)considering the nutritional qualitative and quantitative parameters as a strategy to improve the management of those diseases seems to be decisive. In this context, mitochondria, as major actors of lipid metabolism, represent an obvious target to be explored. Therefore, the present study was designed to evaluate whether a reduction of glucose intake associated to its partial replacement by galactose could slow down the obesity-related adipogenesis through a modulation of the mitochondrial structures, dynamics, and functions. To this end, two nutritional conditions were compared: HG (25 mM glucose) versus LG-GAL (5 mM glucose + 20 mM galactose) in the 3T3-L1 cellular model of differentiating adipocytes. As a peculiar note, preliminary data acquired in our lab evidenced that culturing 3T3-L1 in the LG condition is associated to a drastic reduction of the adipogenic yield (Supplementary Data 1). In such a context, we hypothesized that a poorer adipogenic commitment would further impact the mitochondrial network in correlation to this phenomenon, guiding our experimental strategies with 25 mM monosaccharides concentrations to understand the poor adipogenic influence on our mitochondrial function, structure, and dynamics assessment.

Our findings strongly point out a profound impact of LG-GAL on the adipogenic process in 3T3-L1 at the cellular morphology, subcellular, and molecular levels when compared to our HG referent condition. The cellular morphology assessment evidences a clear reduction in the number and size of lipid droplets, which is highly correlated to a reduction of the percentage of differentiated cells in the LG-GAL condition. To exclude any deleterious mechanism induction in LG-GAL, the cell viability was assessed and clearly indicates a stability in the number of cells all along the differentiation process, demonstrating that 3T3-L1 cells perfectly tolerate this condition. Those observations highlight a direct correlation between the reduction and partial replacement of glucose by galactose with a poorer adipogenic commitment without affecting the cells number. In addition, our results regarding carbohydrates levels clearly demonstrate that 3T3-L1 do similarly and increasingly intake both glucose and galactose as potential manageable nutritional resources. Galactose intake, independent to insulin-mediated stimulation, was not due to insufficient energetical production, since neither intracellular nor extracellular glucose reserves have ever been exhausted. This observation is correlated to a better carbohydrates management in the LG-GAL condition, reflecting a well-controlled physiological carbohydrates intake. In turn, it allowed preventing any abnormal energetical intake that could lead to an excessive lipid storage effect (hypertrophy). In our study, the investigation regarding several anti-adipogenic aspects of LG-GAL was performed with a special focus on separating early differentiation from late maturation phases. PPARγ is a master regulator whose expression is mandatory to induce the adipogenesis. The modulation of its expression is largely described to be a potent strategy to help prevent adipogenesis [54,55]. Glucose-lowering strategies, such as those reached by anti-diabetic drugs, are already known to reduce PPARγ expression [55,56]. In a context of obesity, such a modulation of PPARγ expression represents a very interesting therapeutic angle to reduce obesity-associated WAT pathological remodeling [57,58,59,60]. Our study clearly indicates that the LG-GAL condition has similar modulating effects on PPARγ with a reduction of its expression all over the adipogenesis. To further investigate the impact of LG-GAL on adipogenesis in 3T3-L1 cells, another major regulating actor of the early adipogenic phase, preadipocyte factor-1 (PREF-1), a transmembrane protein expressed in preadipocytes, is commonly presented as a trustful indicator of the adipogenic yield [61]. Indeed, several studies demonstrated that conditions promoting the expression of this factor keep cells under the preadipocyte status and, in this way, slow down the adipogenesis [62,63,64]. This is particularly the case of some nutrients such as some flavonoids, which, by increasing PREF-1 expression, also reduce adipogenesis [65]. In our study, LG-GAL led to a higher PREF-1 gene expression compared to HG in differentiating 3T3-L1, supporting at the molecular level the belief of an impact of the nutrient supply parameter on adipogenesis. This is particularly interesting in an attempt to develop new strategies based on a better nutritional control, since increased PREF-1 expression is also known to improve resistance to high-fat diet, which is a food behavior associated to obesity development and progress [66].

Another critical aspect of adipogenesis-related WAT remodeling is the lipid droplet network expansion, which is a feature that is highly dependent on the expression of mature adipocyte markers. Here, our results clearly indicate a substantial impact of LG-GAL on the maturation phase of adipogenesis. Mature 3T3-L1 adipocytes are characterized by an extensive network of lipid droplets providing a disposable energetical resource. Adipocyte lipid droplets are not “simply” constituted of lipids but are quite complex molecular architectures that are at some point considered as organelles, whose size and shape are under highly regulated mechanisms [46,47,67]. Expected to follow the highly dynamic requirement of adipocyte functions, lipid droplets are surrounded by proteins shaping and enzymatically assisting adipocyte lipogenesis/lipolysis pathways [48,68]. Among the family of lipid droplets-related proteins, perilipin are decisive for adipocyte nutrient storage function. Our data indicate that the use of LG-GAL as a carbohydrates source is associated to a significant reduction of PLIN1 expression in 3T3-L1 cells as compared to the situation in the HG condition. Similar observations were done for another important gene encoding a protein related to adipocyte lipogenic metabolism, the fatty acid synthase (FASN). This enzyme, by catalyzing a major step of fatty acid synthesis, is also essential to ensure lipid droplet network extent in a context of adipogenesis. Interestingly, the reductions of PLIN1 and FASN expression are individually reported as improving resistance to HG-induced obesity and insulin sensitivity and reducing adipose tissue inflammation [69,70]. Finally, the modulatory adipogenic potential of LG-GAL was also evaluated through the expression of adiponectin, which is one of the major adipokines secreted by adipose tissue. Throughout our study, a clear reduction of adiponectin expression in differentiating 3T3-L1 cells was associated to the LG-GAL condition, which is an observation mainly explained by a clear reduction of the number of differentiated cells in this context.

In addition to the adipogenic molecular factors known to be of critical importance in modulating WAT function, another key central actor orchestrating adipogenesis is the mitochondria. In this study, the impact of LG-GAL on the mitochondrial network structure of differentiating 3T3-L1 cells was examined under different points: the distribution area of the network and their size. Our results clearly indicate that the mitochondrial network is a highly dynamic structure following a time- and nutrient-dependent evolution. During adipogenesis, a substantial modulation of the mitochondrial network was noticed in parallel with a significant change in the lipid network on D7. At this stage, an important restriction of the distribution area constrains the mitochondrial network to reorganize, condensate, and even to be less abundant around the swelling lipid droplets. Through our results, we recognize D5 as a key time point of 3T3-L1 adipogenesis regarding the significant mitochondrial network extent in both HG and LG-GAL conditions. This progressive mitochondrial network expansion reaching a significant peak 5 days after inducing adipogenesis seems to be mainly explained by an increase of the mitochondrial number. In addition, a positive effect of LG-GAL on both the distribution and number of the mitochondrial network was noticed. In our study, it also appears that this influence on the mitochondrial network is based on a change of the mitochondrial number rather than their size. Interestingly, those observations are to be compared to substantial changes regarding markers of the mitochondrial biogenesis and dynamics. In our study, LG-GAL was associated to a significant increase of PGC-1α expression over the adipogenesis compared to the HG condition. Next, the impact of the LG-GAL condition was evaluated from the point of view of fusion and fission, which are two linked mechanisms that are strongly controlled and ensuring healthy and adequate mitochondrial function. In a normal physiological status, fission and fusion act at equilibrium. However, in an obese context, it is admitted that this equilibrium gets disrupted and is linked to mitochondrial dysfunction mechanisms in adipocytes [71]. Moreover, fusion and fission mechanisms are well known to be modulated by the nutritional context through regulation by metabolic sensors, which is once again a disrupted mechanism in obese conditions. Through our observations, we demonstrate that LG-GAL tends to globally reduce both the expression of mitochondrial fusion and fission markers in differentiating 3T3-L1 when compared to the HG condition. Higher stimulation of those fusion and fission events in the high-glucose condition potentially points out a long-term mitochondrial dynamics remodeling request in response to high energetical stimulation. This observation is supported by previous studies evidencing that HG levels are associated to considerable mitochondrial stress, ultimately leading to mitochondrial dysfunction [34]. Since mitochondrial stress and dysfunction promote obesity-related adipogenesis [72,73], our study suggests another positive effect of our glucose lowering and replacement strategy.

The last parameter measured in the present study to evaluate the impact of LG-GAL on the mitochondria of 3T3-L1 cells was the integrity of their metabolic function. Our results regarding the expression of key enzymes of the Krebs cycle and mitochondrial respiration strongly indicate that LG-GAL reduces the expression of those catabolic actors in differentiating 3T3-L1. Such an observation can easily be explained by the fact that galactose is not an energy resource for adipocytes and, in this context, it does not lead to an overstimulation of the mitochondrial catabolism, as it is the case in the HG condition. This consideration about the mitochondrial metabolism function was further confirmed by an evaluation of mDIC, which is a protein playing a key role in dicarboxylate exchange between the mitochondria and the cytosol [74,75]. In case of obesity, this increased expression of mDIC is observed in WAT and reflects glucotoxicity and mitochondrial overloads [76]. Our results clearly underline that this carrier is highly expressed in the HG condition and that the LG-GAL condition helps reduce its expression during the early adipogenic phase. This result reinforces a precocious impact of LG-GAL on mitochondria function due to its deep anti-adipogenic effect and its impact on the improvement of WAT remodeling in an obesity context. Finally, the mitochondrial health status investigation assessed by performing a Mitotracker Red CMXRos reinforces the evidence of healthier mitochondria in the LG-GAL condition, since this stain entrance depends on mitochondrial membrane potential. In case of mitochondrial dysfunction, the mitochondrial ETC (electron transport chain) may be impaired, modifying mitochondrial membrane potential and further reducing mitochondrial probe accumulation. Higher mitochondrial staining in the LG-GAL condition (Figure 4) corroborates with less overstimulated and more functional mitochondria than in the HG condition.

As a final note, the overall design of this study was consciously constructed to focus on the cellular and subcellular impact of replacing and lowering the glucose strategy on adipogenesis and mitochondrial function, structure, and dynamics. Using a cellular model of differentiating adipocytes helps specifically focus on the effect of this nutritional strategy on adipocytes driving away systemic metabolism consideration. Nevertheless, the benefit of such a strategy should be further investigated and compared to what was previously observed [35] at the systemic and whole organism scale. At this level, galactose could have undergone a first hepatic pass effect, lowering its blood circulating levels. Such an effect should be deeply investigated to validate the potential beneficial effect of the nutritional strategy we previously discussed. For this reason, evaluating precisely the effect of such a diet change on murine mouse models with a specific focus on the hyperplasia phenomenon could be of great interest. To a larger extent, studying stem cells undergoing adipocytes differentiation in non-adipose tissue such as the muscle is another aspect that should be studied in an obesity context. Finally, a thorough evaluation of the metabolic profile of 3T3-L1 cells could definitely help in better understanding how the adipocytes and mitochondrial function adapt in those two different carbohydrates conditions. In addition, the various tools used in this research to appraise the mitochondrial structure, function, and dynamics globally indicated a significant improvement of the mitochondrial health status in the LG-GAL condition. However, deeper investigations, especially in terms of cellular oxidative stress and metabolic profile changes including mitochondrial respiratory capacities, are highly needed to validate mitochondrial structure, function, and dynamics differences observed among our different experimental conditions. Finally, we hope that our data could constitute a reflexive base for all the actors directly or indirectly working with obese patients health and stimulate the research around diet modulation power and benefits.

## 5. Conclusions

**Figure 7:**
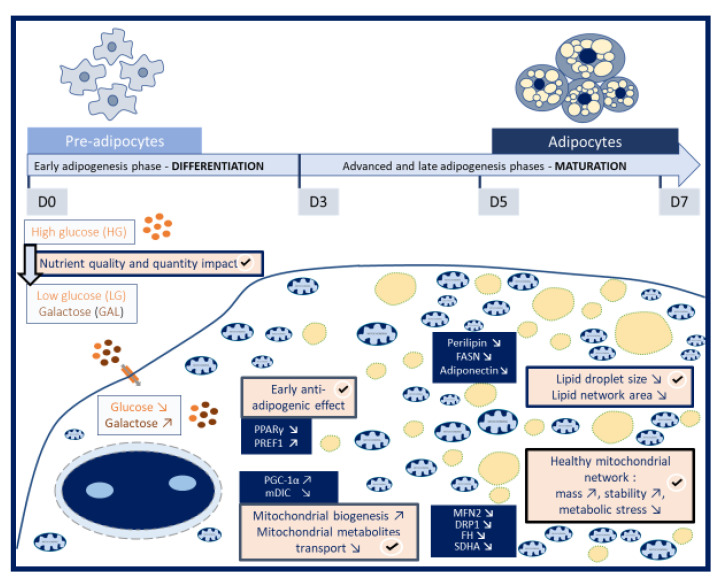
The glucose lowering and replacement by galactose (LG-GAL) strategy during the adipogenic process (7 days) at the differentiation and maturation phases. The differentiation of 3T3-L1 preadipocytes into adipocytes in a standard high-glucose (HG) condition (25 mM) compared to a situation of LG-GAL (5 mM glucose + 20 mM galactose) aims at evaluating the impact of both nutrient quality and quantity to modulate the adipogenic process. Exposing differentiating 3T3-L1 cells to the LG-GAL condition compared to the HG condition leads to decreased intracellular levels of glucose and an important intake of galactose independently on energetical production requirement. Simultaneous carbohydrates intracellular levels and quality modifications do influence adipogenesis at its early phase, as displayed by an anti-adipogenic effect witnessed by early adipogenic marker expression modulation (decreased expression of PPARγ and increased expression of PREF-1). The LG-GAL condition does also impact the early adipogenic phase through influencing the mitochondrial network, as evidenced by an increase in PGC-1α reflecting respectively increased mitochondrial biogenesis. Those major remodeling events occurring during the early adipogenic phase further influence mature adipocyte features acquirement and mitochondrial network evolution during the maturation phase. The early anti-adipogenic effect of LG-GAL is further linked to a reduction of expression of three main markers of mature adipocytes (PLIN1, FASN, and the adiponectin) correlated to a significant reduction of the lipid droplets size and network area during the maturation phase. The glucose lowering and replacement by galactose (LG-GAL) strategy leads to an improvement of the overall mitochondrial health status with an increase of the mitochondrial mass, an increased mitochondrial network stability, and a reduction of the mitochondrial metabolic stress. Through this combination of an anti-adipogenic effect with an overall improved mitochondrial health status, our glucose lowering and replacement by galactose strategy displays promising clues in regard to the research of obesity-controlling strategies.

In conclusion, the impact of LG-GAL on the main actors regulating early adipogenic phases clearly highlight the precocious and decisive prevalence of the nutrient intake parameters on adipogenesis (Figure 7). A modulated expression of key markers of the early adipogenesis phase (PPARγ and PREF-1) in the LG-GAL condition are correlated to a reduced adipogenesis yield. This modulation of the adipogenesis linked to the LG-GAL condition leading to a significant reduction of mature 3T3-L1 adipocytes percentage is sustained by a reduction of the expression of mature adipocytes features (expanded lipid droplet network, de novo lipogenesis enzyme, and adiponectin increased expression). The beneficial effect of LG-GAL as an obesity-controlling and preventing strategy was also clearly demonstrated through its impact on the mitochondrial network of differentiating 3T3-L1 cells. The LG-GAL condition is associated to increased biogenesis, leading to a higher number of healthy mitochondria in differentiating cells. The decreased stimulation of both fusion/fission mechanisms in differentiating 3T3-L1 in the LG-GAL condition witnesses a more stable mitochondrial network, assuming a healthier mitochondrial status. The LG-GAL condition, by avoiding excessive energetical supply, leads to a decreased mitochondrial catabolism (Krebs cycle and respiration) and dicarboxylates transport. Through this combination of an anti-adipogenic effect with an overall improved mitochondrial health status, our nutritional strategy offers encouraging perspectives. Modulating both the quality and the availability of nutritional supplies appears to be a preliminary step to any further therapeutic considerations. Finally, this work also gives rise to the significance of the “nutritional programming” concept to prevent obesity with a beneficial effect of lowering and replacing glucose during key developmental phases involving adipose tissue development.

## Supplementary Materials

The following are available online at https://www.mdpi.com/2072-6643/12/10/2984/s1.
