# Supplementary material for: Influence of Nutritional Intake of Carbohydrates on Mitochondrial Structure, Dynamics, and Functions during Adipogenesis
Source: Nutrients. 2020 Sep 29;12(10):2984. doi: 10.3390/nu12102984 (PMC7600802; doi:10.3390/nu12102984)
Supplement: Supplementary file 1 [file nutrients-12-02984-s001.pdf]

Supplementary Data 1

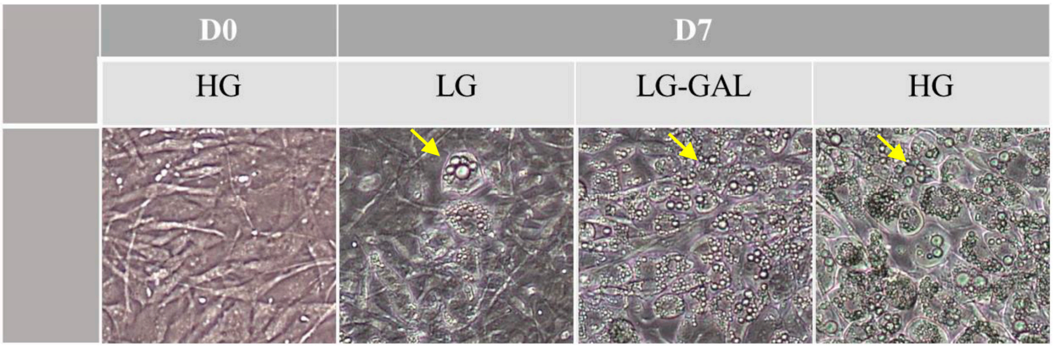

Supplementary Data 1: Representative bright field micrographies of 3T3-L1 cells before (D0) and after (D7) adipogenesis in *HG vs LG vs LG-GAL* conditions. Yellow arrow indicates differentiating cells
